# Efficacy Analysis of Selection of Distal Reference Point for Tibial Coronal Plane Osteotomy during Total Knee Arthroplasty: A Literature Review

**DOI:** 10.1111/os.13054

**Published:** 2021-06-17

**Authors:** Jie Men, Han‐guang Liang, Zhi‐wei Wang, Peng Sun, Wei Feng

**Affiliations:** ^1^ Department of Bone and Joint The First Hospital of Jilin University Changchun China

**Keywords:** Coronal alignment, Osteoarthritis, Osteotomy, Tibia, Total knee arthroplasty

## Abstract

Total knee arthroplasty is an effective treatment for end‐stage knee osteoarthritis. The tibial platform osteotomy must take full account of the coronal plane, the sagittal plane, and the rotational alignment of the tibial prosthesis. During surgery, individual differences in the coronal alignment of the tibia need to be taken into account as poor alignment after surgery can lead to rapid wear of the tibial platform, reducing the longevity of the prosthesis and adversely affecting quality of life. Intraoperative tibial osteotomies are often performed using extramedullary alignment. When an extramedullary alignment approach is used, the proximal tibial osteotomy guide is usually placed in the medial third of the tibial tuberosity. There is no consensus on the most reliable anatomical landmarks or axes for achieving distal tibial coronary alignment. Anatomical points or reference axes that are highly reproducible and precise need to be identified. From available data it appears that most surgeons use the extensor hallucis longus tendon, the second metatarsal, and the anterior tibial cortex to determine the distal localization point. However, its accuracy has not been confirmed in clinical and radiographic data, and the alignment concept and preoperative planning for total knee arthroplasty has paid more attention to rotational alignment, but there are few studies on the coronal alignment of the tibia. This article reviews the recent use of the distal tibial coronal osteotomy reference point in total knee arthroplasty. However, due to there being only a small number of studies available, the evidence collected is insufficient to prove that a certain reference axis has obvious advantages and a combination of different reference points is needed to achieve the ideal lower extremity force line angle.

## Graphical abstract

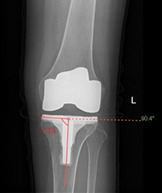

## Introduction

Total knee arthroplasty (TKA) can restore knee function, reduce pain, and improve quality of life for patients with end‐stage osteoarthritis of the knee. About 95% of implants survive for more than 15 years^1^. Prosthesis survival is closely related to alignment of lower limbs after TKA. Good lower extremity alignment is an important factor in the success of surgery^2^. Poor lower extremity alignment increases lower extremity stresses, leading to accelerated wear of polyethylene, aseptic loosening, patellar dislocation, and joint instability. This adversely affects postoperative knee function and prosthesis longevity, and ultimately results in failure of the TKA.

**Fig. 1:**
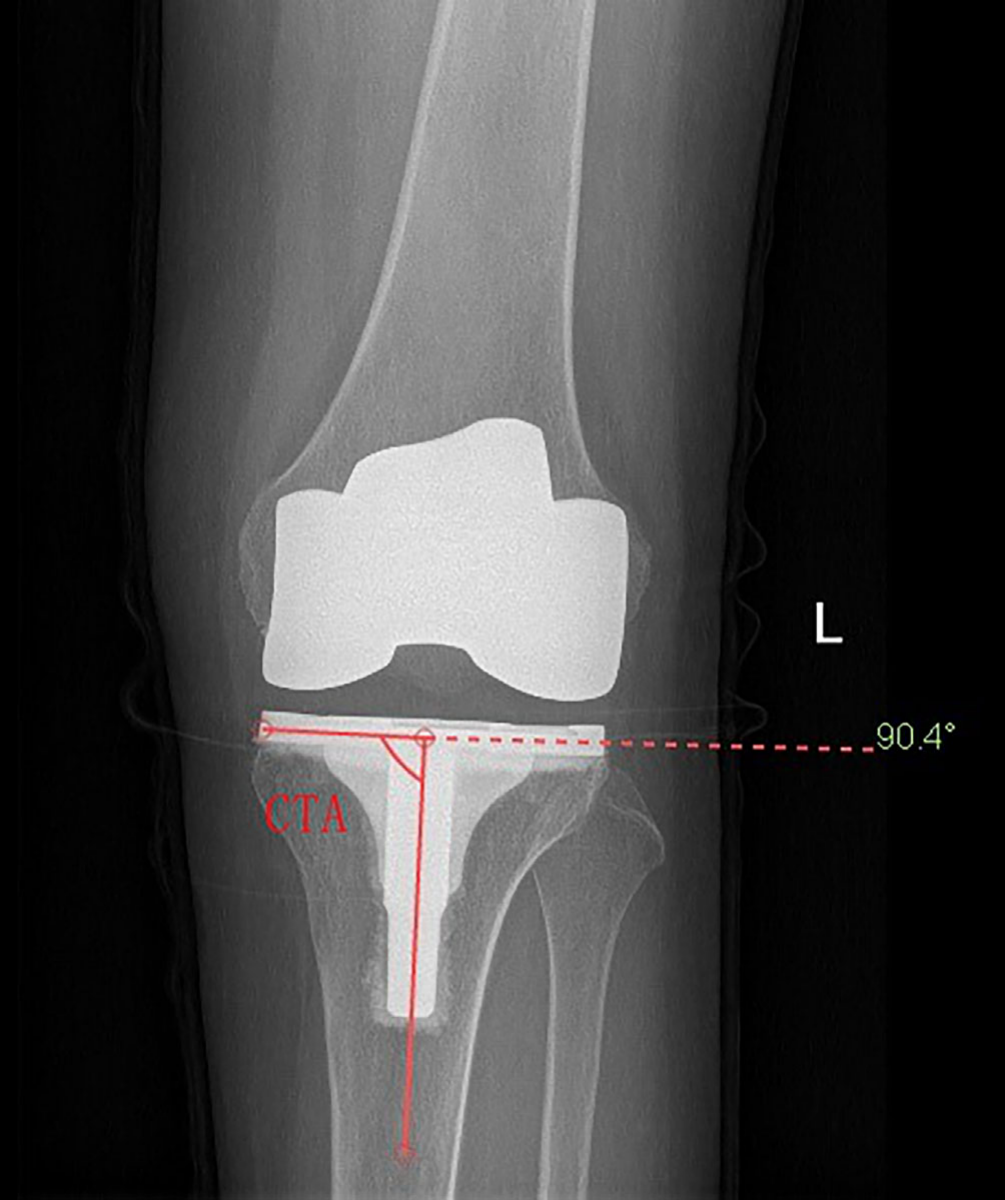
The coronal tibial component angle (CTA) is the medial angle between the mechanical axis of the tibia and the horizontal axis of the tibial tray.

There is general agreement among surgeons that the prosthesis should be aligned parallel to the tibial mechanical axis with deviation of no more than ±3°, to provide a consistent load distribution at the prosthesis–bone interface and to prolong survival of the prosthesis^3^, ^4^ (Fig. 1). Intramedullary alignment and extramedullary alignment are the most commonly used techniques for TKA, with both methods having advantages and disadvantages. Extramedullary alignment is preferred for tibial prostheses because intramedullary alignment is associated with risk of fat embolism and of inversion of the prosthesis and, moreover, cannot be used in patients with tibial deformity, medullary stenosis, or post‐traumatic arthritis^5^.

For extramedullary alignment of the tibia, the current method uses an axis connecting the proximal tibial center to the center of the ankle joint. Therefore, correctly determining the center of the ankle joint is crucial. To align the extramedullary guides to the mechanical axis of the tibia, a number of bony and soft tissue marker points have been proposed as distal tibial reference points; these include the second metatarsal^6^, the extensor hallucis longus tendon (EHLT)^7^, ^8^, ^9^, the tibialis anterior tendon (TAT)^10^, the Achilles tendon (AT)^11^, the extensor digitorum longus tendon (EDLT)^11^, and the dorsal pedal artery (DPA)^12^. In addition, in recent years, the lines connecting the proximal and distal thirds of the anterior tibial margin^13^ or the medial third of the patellar ligament insertion and the distal quarter of the anterior tibial margin have been used a reference axes during tibial osteotomy^14^.

The current clinical use of coronal tibial osteotomies is mostly based on the surgeon's surgical experience and intraoperative empirical judgment, there is no uniform standard for tibial osteotomies, and individual differences in tibial morphology can also have an impact on tibial osteotomies.

Computer navigation techniques can help achieve a more precise line of force in the lower limbs. These techniques allow the surgeon to use sensors to determine the position of the mechanical axes during surgery and achieve precise mechanical positioning. However, high cost, long operating time, and the steep learning curve are reasons preventing wider adoption of computer navigation^4^, ^15^. Preoperative planning and three‐dimensional (3D)‐printed individualized prostheses can reduce the operative time, but the method does not appear to be significantly better than conventional surgery in restoring the lower limb force line; the need for additional imaging studies such as computed tomography (CT) or magnetic resonance imaging (MRI) and the high cost of 3D printing equipment are major limitations^16^.

The purpose of this study was to summarize the advantages and disadvantages of the commonly used coronal anatomical reference points or reference axes in clinical practice, and compare their accuracy, so as to facilitate surgeons to improve the accuracy of postoperative force lines.

## Search Methods

**Fig. 2:**
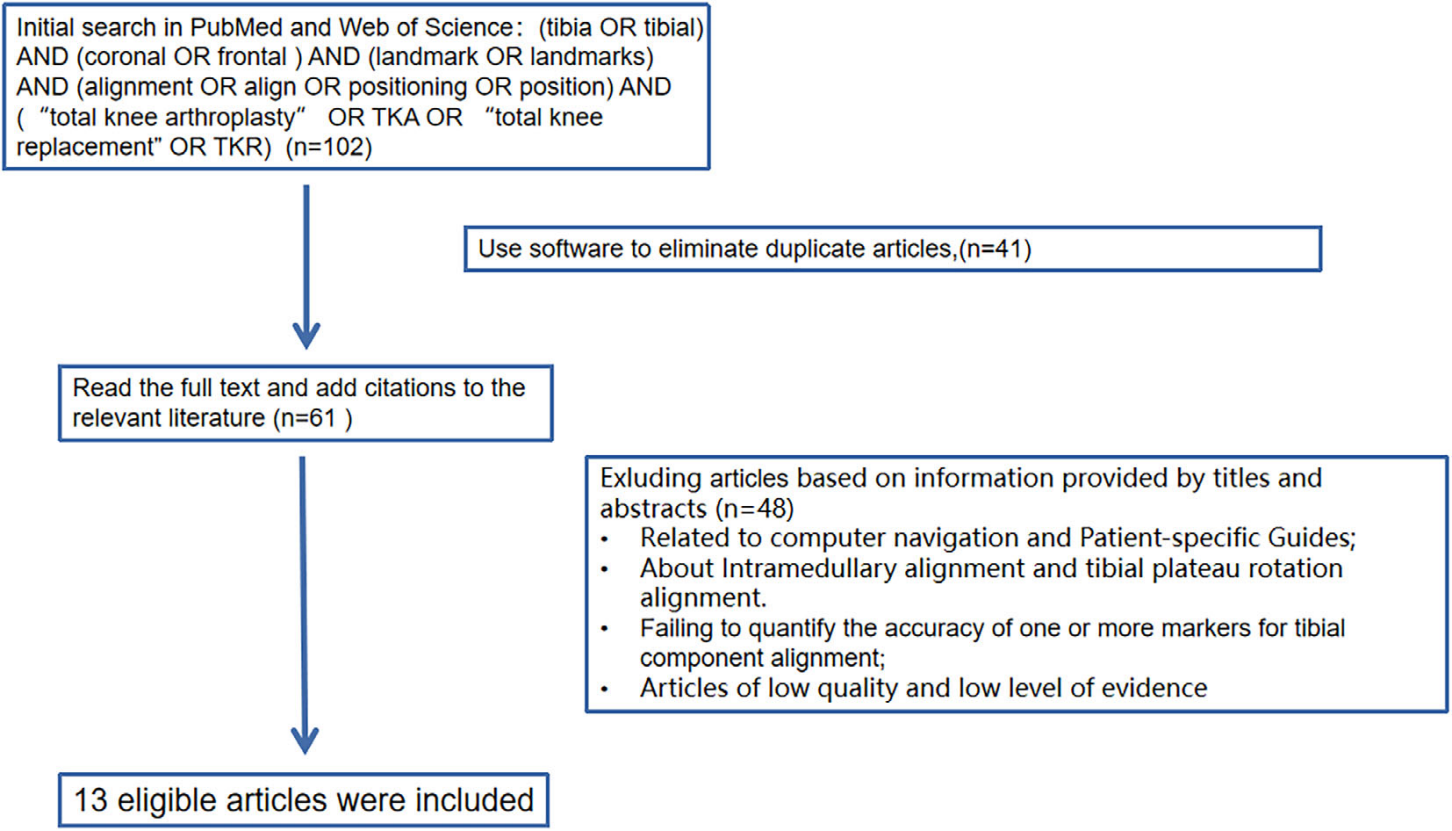
The selection flow for included studies in this review. The first screening step is using softwave to eliminate duplicate articles, and 41 articles were excluded. The second screening step is to exclude according to information provided by titles and abstracts (*n* = 48). Finally, 13 articles were included.

A literature search was conducted using Medline (PubMed) and SCI for articles published between June 2003 and June 2020 on the coronal alignment of tibial components in TKA. The searches were for descriptors “tibia OR tibial,” “coronal OR frontal,” “alignment OR align OR positioning,” “landmark OR landmarks,” and “total knee arthroplasty OR total knee replacement” in combination with using the Boolean connector “AND.” The inclusion criteria for the literature were as follows: (i) basic and clinical research related to the above keywords; (ii) literature types are monographs, papers, guidelines, or reviews. The exclusion criteria for the literature were as follows: (i) related to computer navigation and patient‐specific guides; (ii) about intramedullary alignment and tibial plateau rotation alignment; (iii) fail to quantify accuracy of one or more landmarks for tibial component alignment; (iv) articles of low quality and low level of evidence. Finally, 13 eligible articles were included (Fig. 2).

### Operative Technique

**Fig. 3:**
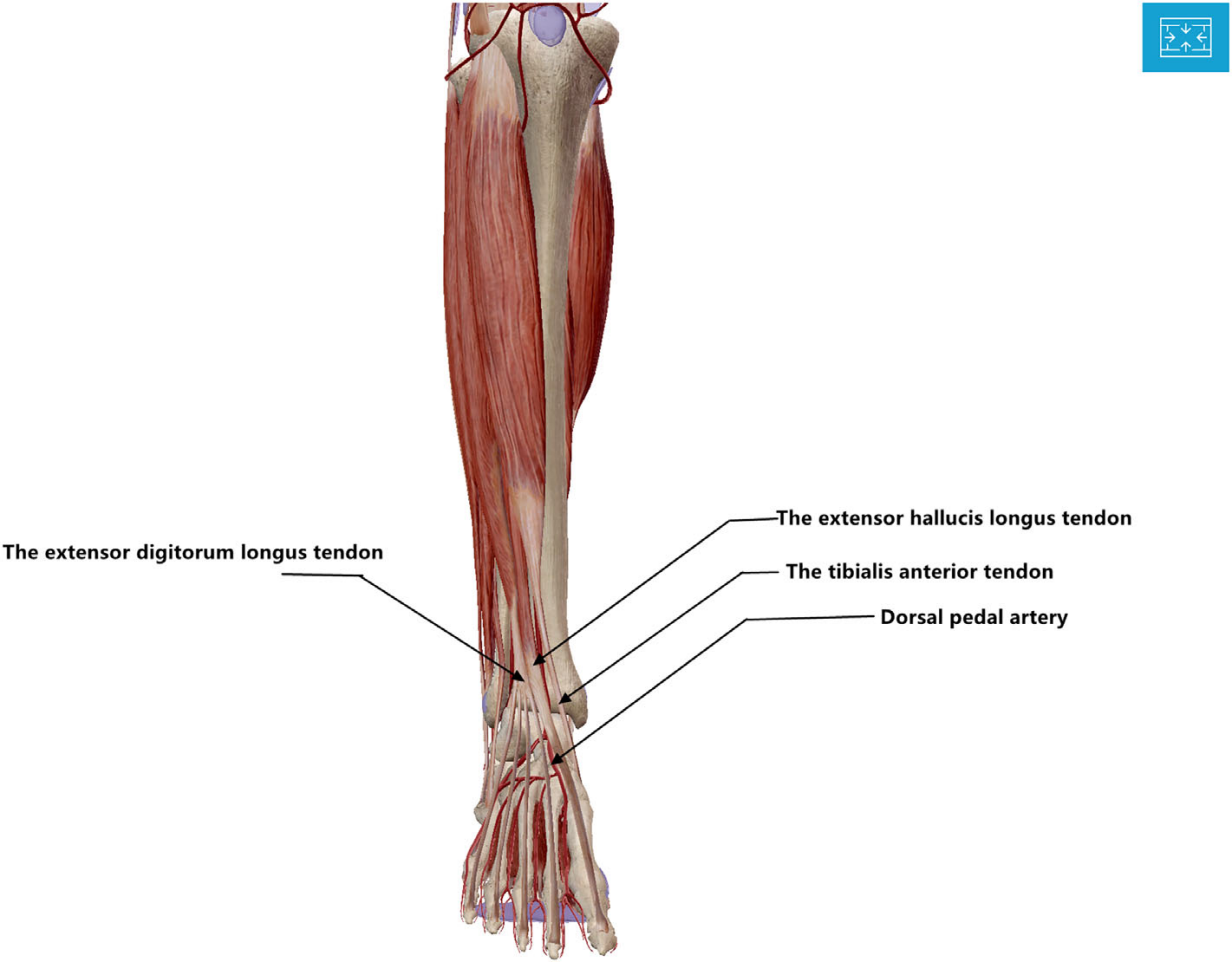
Distal tibial reference point: the extensor hallucis longus tendon (EHLT), the tibialis anterior tendon (TAT), the extensor digitorum longus tendon (EDLT). Dorsal pedal artery (DPA).

**Fig. 4:**
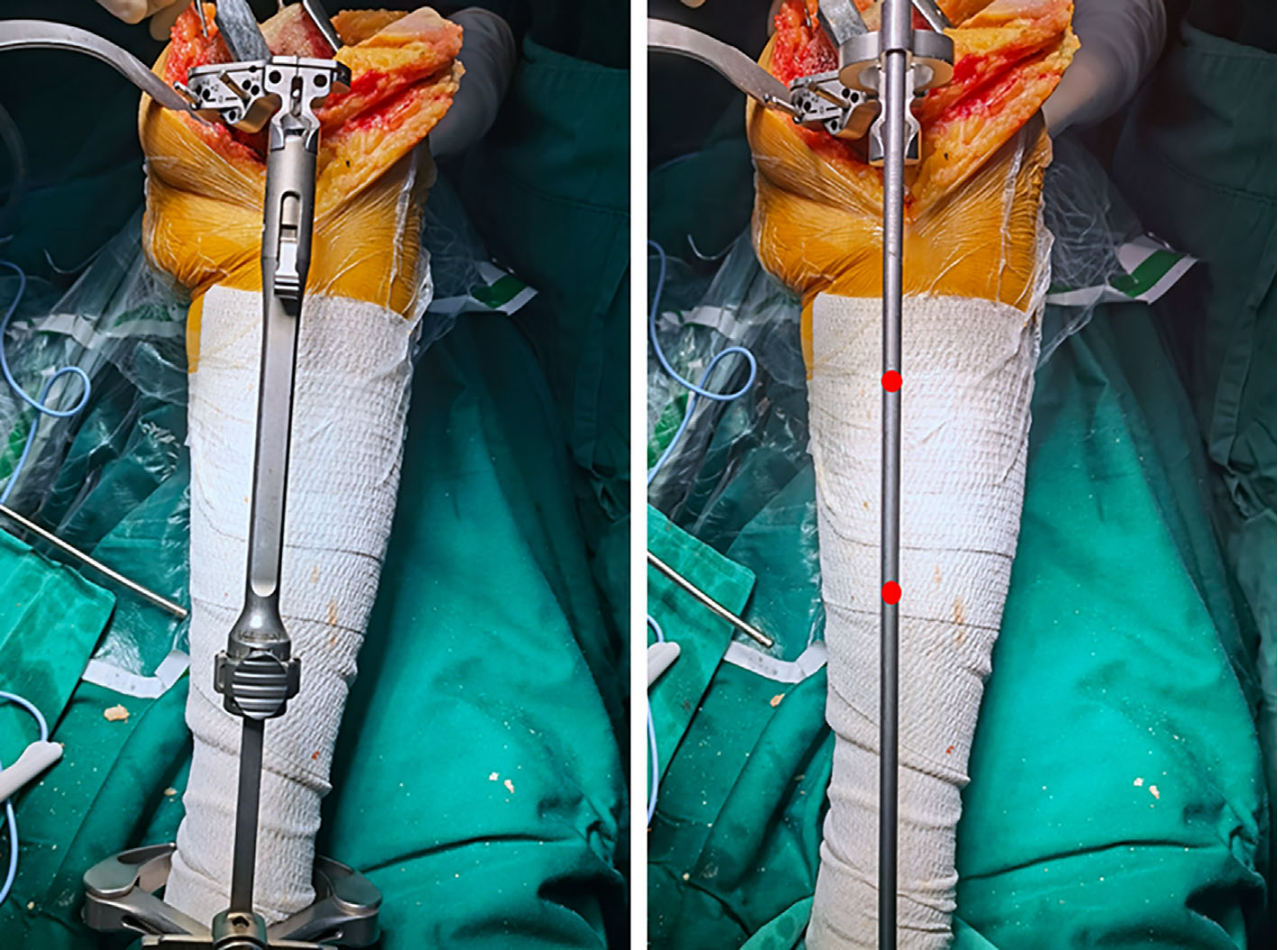
The proximal and distal thirds of the anterior tibial margin

**Fig. 5:**
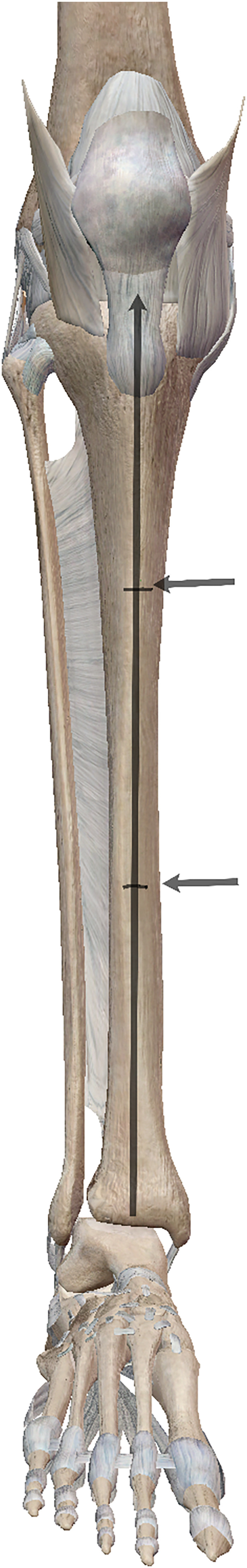
The proximal and distal thirds of the anterior tibial margin. The tibial pole shouldbe parallel to the black line.

**Fig. 6:**
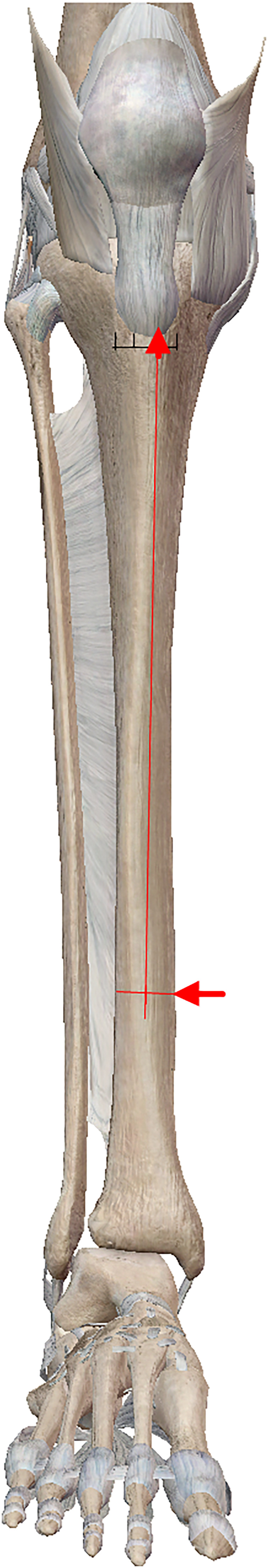
The medial third of the patellar ligament insertion and the distal quarter of the anterior tibial margin. The tibial pole should be parallel to the red line.

The patient laid supine on the operating table after general anesthesia and urethral catheterization, inflatable tourniquet applied preoperatively. The surgical area was disinfected with iodophor, sterile surgical sheet was laid, and after wearing sock, the surgical area was covered with antibacterial film. Using a para‐median incision, the patella was everted after releasing the soft tissues, the medial and lateral menisci and anterior‐posterior cruciate ligaments were excised. The knee was flexed at 90° and the tibia was semi‐dislocated forward. The tibia was osteotomized using an extramedullary osteotomy technique. The osteotomy guide plate was fixed on the proximal tibia and a tibial positioner was installed. The AP axis connected the center of the posterior cruciate ligament at the tibial attachment and the medial one‐third of the border of the patellar tendon at the tibial attachment. The direction of rotation of the proximal end of the guide was in the AP axis. The distal end of the tibial locator should point toward the distal tibial reference point. Reference points were commonly used in clinical practice including the second metatarsal, the extensor hallucis longus tendon (EHLT), the tibialis anterior tendon (TAT), the extensor digitorum longus tendon (EDLT) (Fig. 3), the lines connecting the proximal and distal thirds of the anterior tibial margin (Figs 4 and 5), or the medial third of the patellar ligament insertion and the distal quarter of the anterior tibial margin (Fig. 6). The osteotomy was performed with a pendulum saw after fitting a tibial locator. The osteotomy was determined according to the preoperative X‐ray measurement. The tibial osteotomy surface in the sagittal plane was maintained with a 5° posterior inclination. After completion of tibial osteotomy, femur osteotomy was performed. The femur was osteotomized using the intramedullary alignment technique. Installation of intramedullary positioning rod was carried out. The distal femoral cutting block was then attached to the alignment guide, with adjustment for the anatomical valgus angle of the femur. Finally, four‐in‐one osteotomy and intercondylar molding were performed. Flexion and extension gaps were checked and equalized with the tensor device. Trial prosthetic was implanted. Check the range of motion of the knee, joint stability, implant rotation, patellar tracking, and the lower extremity lines. Finally, the prosthesis was fixed with bone cement, the excess bone cement was removed. Washing the surgical area with saline solution. The tourniquet was loosened and the incision was sutured layer by layer.

### Tendons

The tibial mechanical axis is defined as the line connecting the midpoint of the proximal tibia and the midpoint of the talus. Therefore, it is important to accurately identify the center of the talus when applying an extramedullary guide during a TKA tibial osteotomy. Tendons located just beneath the skin are easily identified during the course of the surgery, without need for assistive devices. Therefore, the TAT, the EHLT, the EDLT, and the AT at the ankle could serve as distal reference points (Fig. 3). The TAT and EHLT are 6.9 and 5 mm wide, respectively. The TAT is located at least 1 cm medial to the center of the talus. The EHLT is closer to the center of the talus at the ankle level than the TAT and so would be the more accurate reference point^8^, ^9^.

In a study based on an analysis of ankle MRIs, Rajadhyaksha *et al*. found the mean distance from the midpoint of the distal tibia to the TAT to be 1.89 mm (1.45–2.33 mm); in 84.4% of ankles, the distance was ≤2 mm^10^. In a cadaver study, Schneider *et al*. examined the horizontal distance of the EHLT and TAT from the midpoint of the talus and also analyzed the anatomical relationship of the two tendons to the internal and external ankle in the neutral position and the pronation and supination positions. The effect of pronation on the distance from the EHLT and TAT to the internal and external ankle was found to be smaller (distance of EHLT tendon, 0.2–0.6 mm; distance of TAT, 2.9–3.5 mm). The deviation of the EHLT was greater than that of the TAT during supination (9.2–10.5 mm *vs* 6.2–6.8 mm, respectively). Using MRI images of healthy volunteers, Schneider *et al*. showed that extreme supination resulted in a deviation of 7–10 mm both TAT and EHLT. The effect of rotation on EHTL was greater in living volunteers than in the cadaveric samples, probably due to the removal of the support band in the latter^9^.

Using ankle joint MRI images of 53 volunteers, Tiftikçi *et al*. compared the distance of the midpoints of TAT, EHLT, DPA, and AT from the center of the talus at a level 2 mm from the distal tibial articular surface and 2 mm from the talar articular surface. The AT was anatomically closest to the center of the ankle joint. Statistical analysis showed that the AT,

the DPA, and the EHLT were significantly closer to the center of the ankle joint^11^. However, it should be noted that the study sample did not include patients with osteoarthritis and tibial torsion deformity.

The EHLT begins at the level of the lower third of the tibia and ends at the distal phalanx of the toe. The EHLT, EDLT, and TAT dorsiflex the ankle. The TAT is most obvious in plantar flexion. To avoid confusion between the EHLT and EDLT during the surgery, the EHLT can be identified preoperatively by active and passive dorsiflexion and plantar flexion of the ankle and marked before the patient is anesthetized. The TAT is the easiest to palpate; even in obese patients, the TAT can be palpated under anesthesia. However, it is located too medially. When the distal reference point of the tibial osteotomy is too medial, postoperative tibial prosthesis valgus deformity may result. The AT is located posterior to the ankle, and existing extramedullary alignment devices cannot be applied directly intraoperatively.

Patients with end‐stage osteoarthritis of the knee may also have developmental joint deformities such as bunion deformity, subtrochanteric deformity, and forefoot and hindfoot deformities. Such deformities also affect the travel of the tendons^17^, ^18^.

Hino *et al*. examined how TAT, EHLT, Akagi line angle, femoral tibial angle (FTA), hallux valgus angle (HVA), and knee valgus angle (KVA) on MRI images were related to the forefoot deformity index of 61 patients with knee osteoarthritis. EHLT, FTA, HVA, and KVA were not found to be related to the severity of deformity and tibial torsion^8^. The study validated the possibility of using the EHLT as the distal reference point to achieve extramedullary alignment.

There were no studies on the use of EHLT, TAT, AT, and EDLT as markers for determining postoperative lower extremity force lines, prosthesis alignment, or outcomes of distal tibial osteotomy. The use of tendons for these purposes needs further study.

### Second Metatarsal

**Fig. 7:**
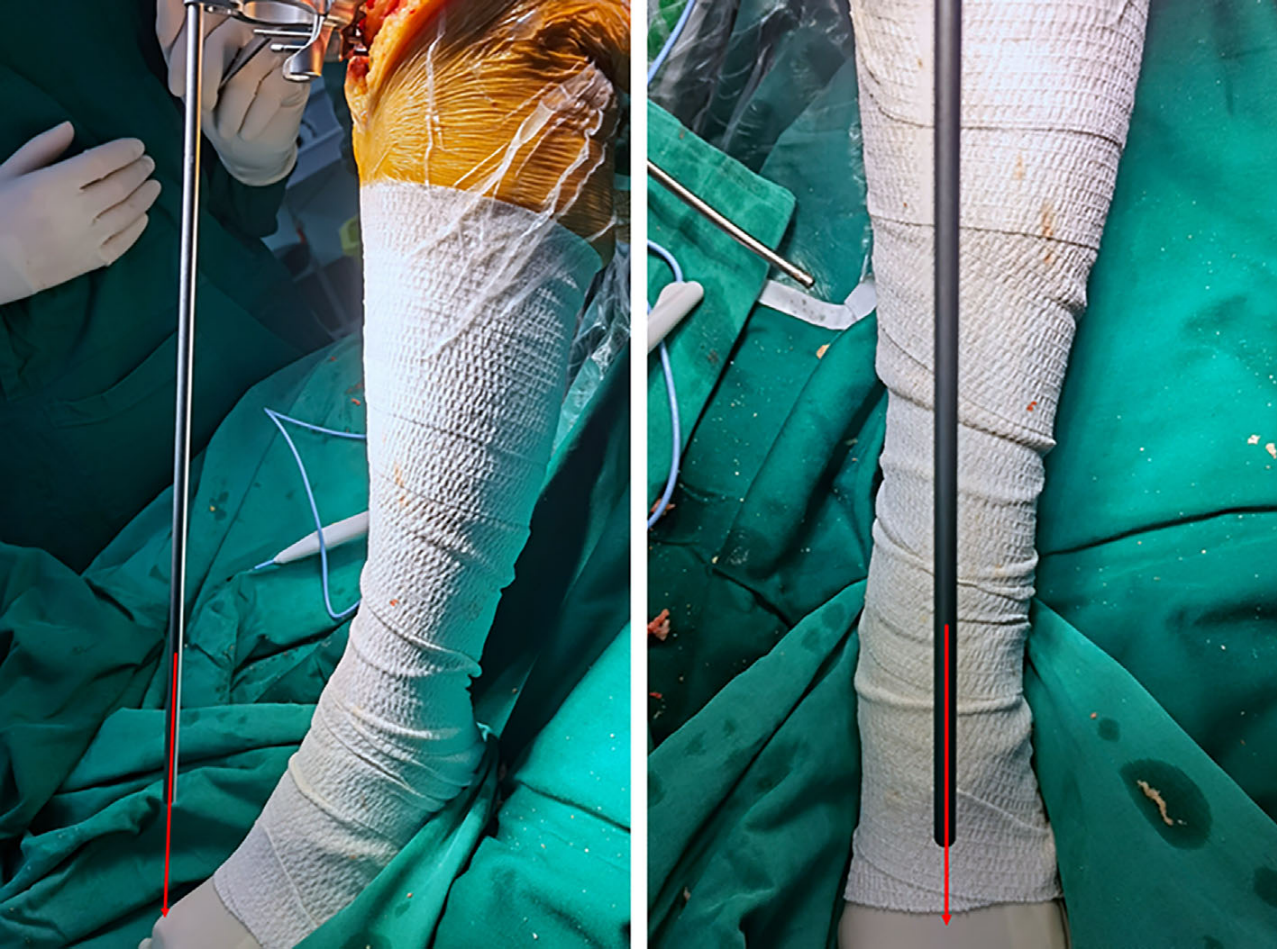
The red arrow should point toward the second metatarsal.

The manual for the use of TKA surgical instruments recommends that the distal end of the tibial cutting block's locating rod should point toward the second metatarsal when extramedullary alignment examination is performed Fig. 7).

**Fig. 8:**
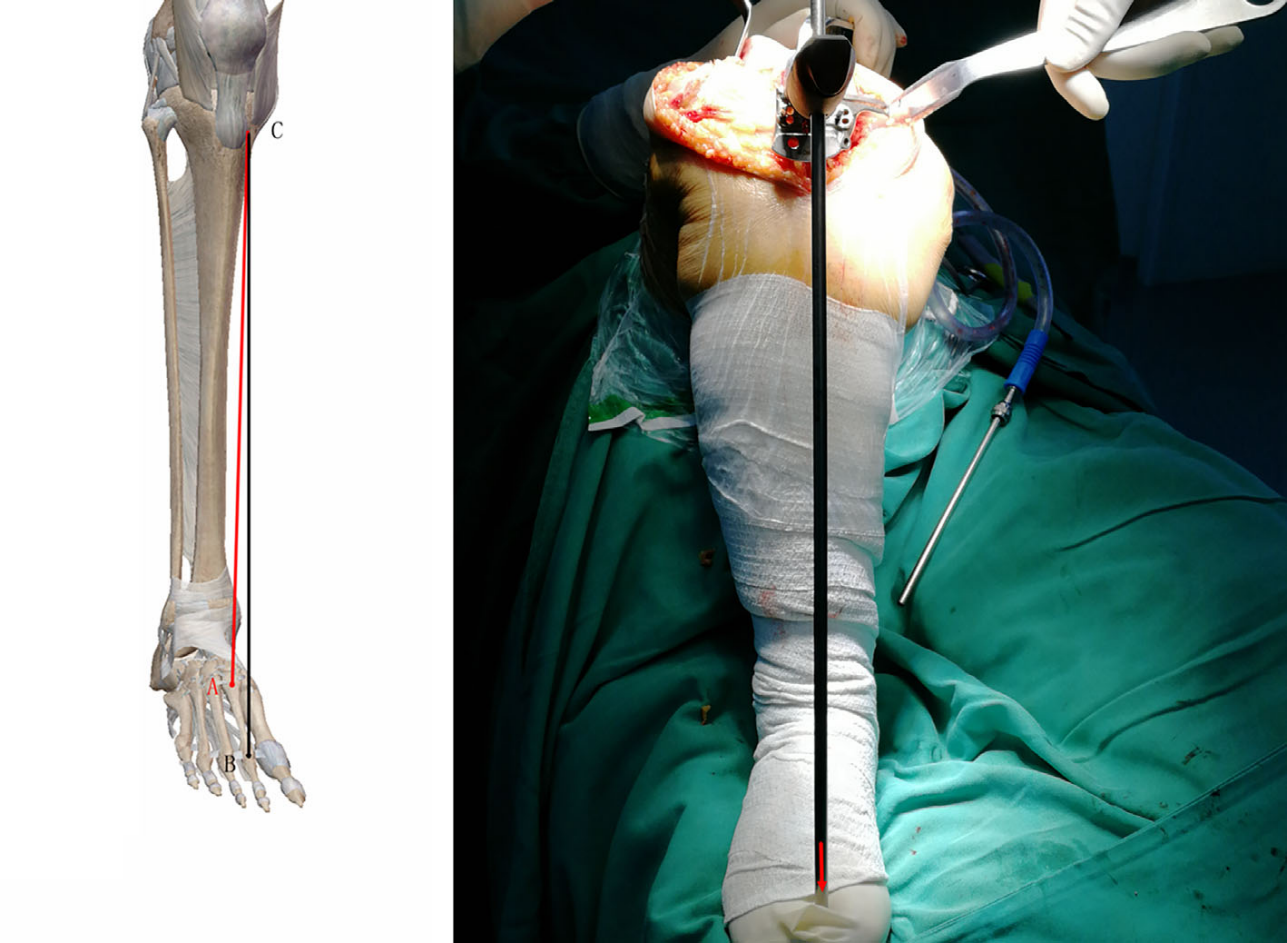
The red arrow should point toward the second metatarsal, A: the base of second metatarsal, B: the distal metatarsal, C: the medial edge of the patellar tendon attachment.

Tsukeoka *et al*. studied a sample of patients with osteoarthritis and rheumatoid arthritis.^6^ The angle between the projected lines of the mechanical axis of the tibia and the longitudinal axis of the tibial cutting guide in the plane perpendicular to the Akagi line and the mechanical axis of the tibia was measured. ±3° as meeting the criteria, when the base of second metatarsal (Fig. 8A) and the distal metatarsal (Fig. 8B) were used as reference points, resulting in a statistically significant difference in the number of extremes between the two groups. There was no significant difference in the rheumatoid arthritis group, and the prosthesis alignment error showed significant positive correlation with the angle between the second metatarsal and the Akagi line^6^. The authors recommended the use of the base of the second metatarsal as a reference point. However, it should be noted that the preoperative neutral ankle position in this article may be different from the intraoperative position. Intraoperative rotation of the ankle joint, both pronation and supination, can affect the accuracy of the tibial cut. This is especially true in rheumatoid arthritis with ankle joint involvement. Therefore, it is not advisable to use the second metatarsal as the only distal marker for extramedullary positioning of the tibia.

Rotational errors occur mainly in the proximal tibia and increase with the distance between the bone and the locating bar^19^. This is why the abnormality rate is significantly reduced when the positioning rod is pointed toward the base of the second metatarsal. The use of the second metatarsal as a reference point is an improvement on the existing extramedullary guidance system: it does not increase the cost of the procedure, does not prolong the operation time, and can be used with all existing extramedullary guidance systems. However, with the use of a tendon as a reference point, accuracy can be affected by ankle position and pedal deformity.

### Dorsal Pedal Artery

The DPA is a continuation of the anterior tibial artery in the front of the ankle. It is located deep to the EHLT and travels along its lateral aspect, adjacent to the approximate center of the ankle joint (Fig. 3). The DPA can be palpated at the most prominent part of the dorsal aspect of the navicular bone. Sugimura *et al*. used Doppler ultrasound to identify the exact location of the DPA. In patients with osteoarthritis and rheumatoid arthritis, the location of EHLT and DPA may be closer to the center of the ankle: the DPA may be 0.4 ± 3.4 mm more laterally located, the EHLT 0.7 ± 3.5 mm more medially located, the EDLT 5.7 ± 3.1 mm more laterally located, and the TAT 12.0 ± 3.3 mm more medially located^12^. Dorsiflexion of the foot increases skin tension and obscures the pulsation of the DPA. Stretching of the skin during plantar flexion may also make palpation of the DPA pulsation difficult^20^. In addition, the DPA pulsation is not palpable in 3.1%–13.8% of healthy individuals^21^ and may be diminished or absent in obese patients and in those with atherosclerosis and peripheral vascular disease^20^. Intraoperatively, wrapping of multiple layers of sterile towel sheets around the ankle and the use of inflatable lower extremity tourniquets makes location of the DPA difficult. In contrast to the DPA, the location of the EHLT and TAT can be marked either before or after tourniquet inflation, and then easily identified during surgery. Thus, the DPA should not be used as the only distal marker for extramedullary alignment of the tibia.

### Medial and Lateral Ankle Midpoints

**Fig. 9:**
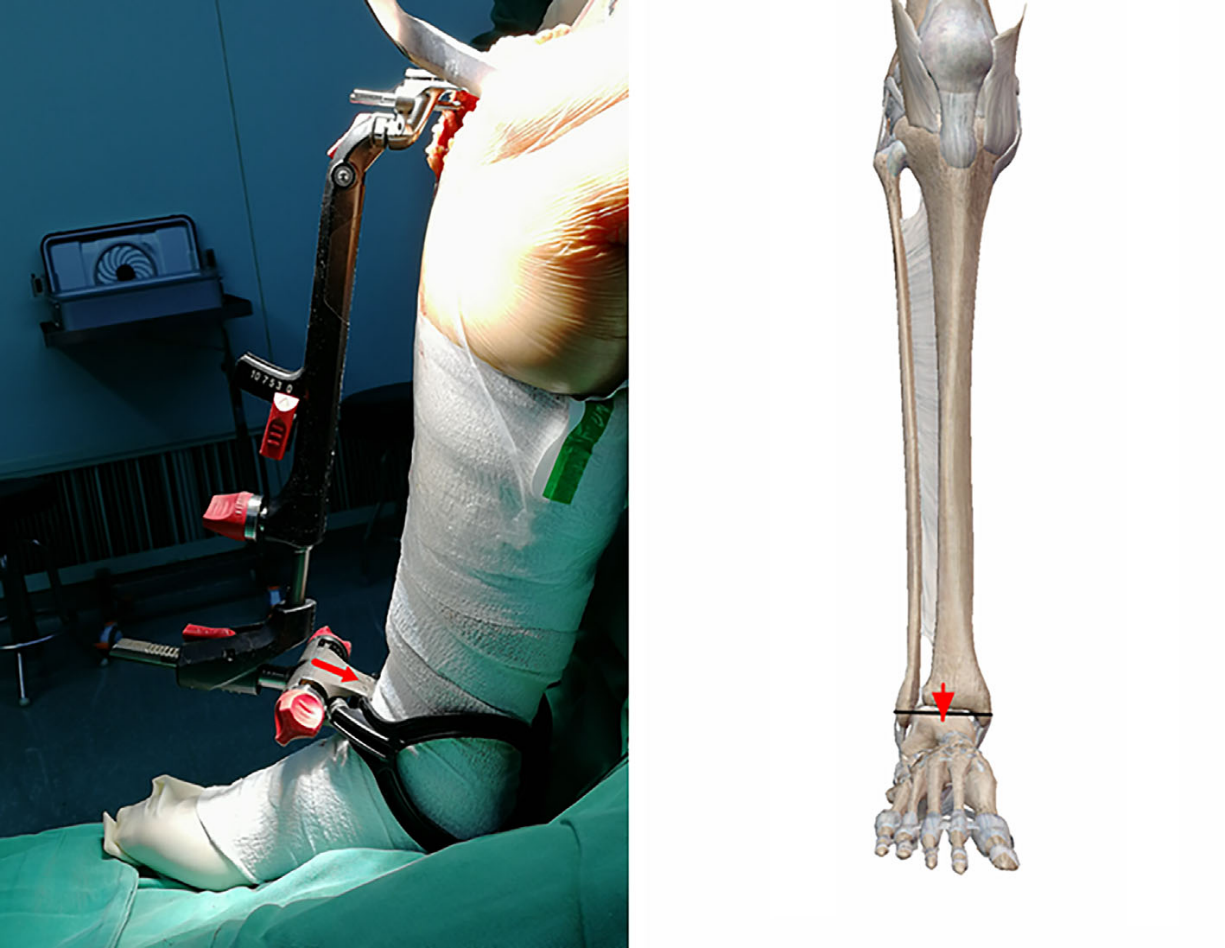
The red arrow should point to the medial and lateral ankle midpoints.

The tibial extramedullary guided bilateral ankle technique is used because the center of the medial and lateral ankle is close to the midpoint of the talus (Fig. 9). The ankle center is located 3–5 mm medial to the midpoint of the line connecting the medial and lateral ankles^14^. According to Tsukeoka *et al*., the distance between the soft tissue center and the true ankle center is only 4.5 mm at the most^22^.

The ankle clip of the extramedullary guide is fixed to the distal tibia, but it does not capture the most medial point of the medial ankle and the most lateral point of the lateral ankle, so it is unclear how much lateral error should be considered in determining the center of the ankle, the disadvantage of which can be difficult during surgery because the sterile sheet is wrapped to locate the medial 3–5 mm. Shigeki analyzed CT data, found that the mean offset distance with reference to the tibial AP axis was 1.8 ± 0.9 mm medial to the intermalleolar midpoint on the coronal plane. The intermalleolar midpoint in the coronal plane is a reliable landmark for the ankle center during TKA^23^.

Another article similarly confirmed that the tibial mechanical axis was approximately 2 mm medial to the center of the ankle on anteroposterior view. Tibial mechanical axis marked on the skin could be referred to osteotomize the tibial plateau^24^. However, the ankle clip of the extramedullary guide is not affected by the movement of the ankle.

## Anterior Tibial Border

The tibia is trigonous in shape. The anterior edge of the tibia and the medial aspect are covered only by skin. It is therefore a convenient anatomical marker for extramedullary alignment. Unlike the bony and soft tissue markers around the ankle, the tibia is not affected by ankle deformity or the presence of excessive soft tissue in obese patients.

The two main tibial reference lines are the line connecting the proximal third and the distal third of the tibial anterior margin^13^, and the line connecting the medial third of the patellar tendon and the distal quarter of the tibial anterior margin. Both of these lines were found to be almost parallel to the mechanical axis in the study by Fukagawa *et al*., where they analyzed CT scan data using 3D imaging software and measured the angles made by 10 axes to the mechanical axis in the coronal and sagittal planes^25^.

The median line connecting two points located in the middle of the tibia and 5 mm proximal to the ankle joint, and a line at 58% and 90% of the length of the tibia, are the most significant anatomical references parallel to the mechanical axis of the tibia^26^.

In a study by Gianluca Cinotti *et al*., the tibial crest cannot be considered as a primary anatomical landmark for the coronal alignment of the tibial component in Caucasian patients. In cases where it is difficult to improve the alignment of a total knee arthroplasty using multiple reference points, the axis of the proximal body projection of the tibial mechanical axis connected to a point 23% of the length of the tibia can be used as a reference^27^. In the other article, Tadashi *et al*. demonstrated that the tibial crest in the coronal planes could be useful guidelines in performing TKA, even if the patient has tibial bowing or severe varus deformity^28^.

Proximal reference points (such as the medial edge of the patellar tendon) are too medial, and so tend to result in internal valgus deformity. Similarly, the distal reference points (midpoint of the tibial tuberosity and proximal quarter of the tibia) are too lateral, and so lead to valgus deformity.

Osteotomy performed using only marker points at the anterior tibial margin may be influenced by the physiologic torsion of the tibia and the thickness of the subcutaneous soft tissues, and so the extent of the tibial deformity should be preoperatively determined on tibial orthotopic radiographs.

In a study of tibial torsion, the center of the ankle was found to be shifted 9–11 mm laterally relative to the proximal tibial axis. The anteroposterior axis of the ankle (i.e., anterior projection of the center of the talus) was on average 19°–27° externally rotated compared to the proximal tibial anteroposterior axis (the line connecting the insertion of posterior cruciate ligament of the tibial platform and the medial edge of the tibial tuberosity)^29^.

Trigonometric analysis of the effect of different rotational alignments of the proximal and distal anteroposterior axes on the coronal plane of the tibial osteotomy showed that a 2 mm external displacement of the distal end resulted in 0.88° of internal rotation of the osteotomy, implying that a 10 mm external displacement would result in 4.4° of internal rotation of the tibial osteotomy. To compensate for the external rotation of the ankle and to avoid tibial osteotomy with internal rotation of the incision, the distal end of the extramedullary guide should be internally relocated; however, it is not clear how much internal displacement is required^19^.

Assuming a tibial length of 160 mm (160 mm × tan (1°) = 5.25 mm), a difference of approximately 5 mm in the horizontal plane would result in only a 1° change in coronal plane alignment, and an error of more than 15 mm in the horizontal plane would result in a 3° deviation in alignment with the mechanical axis of the limb.

Because the proximal tibial cut involves coronal and sagittal alignment, the internal rotation is further exacerbated if the extramedullary guide is laterally displaced distally, as the posterior inclination of the tibial osteotomy changes to a posterior medial tibial slope in this case.

In TKA surgery, rotational mismatch between the proximal tibia and the ankle has significant impact on the accuracy of the proximal tibial cut^29^. Rotational errors occur primarily in the proximal tibia and increase with the distance between the bone and the positioning bar^19^. The closer the distal tibial reference point is to the proximal tibia, the greater the error in the coronal plane angle. Therefore, to reduce the error, a reference point that is distal to the bone surface is preferred.

### Effect Analysis

The coronal tibial component angle (CTA) is the medial angle between the mechanical axis of the tibia and the horizontal axis of the tibial tray on the AP radiographs. CTA on the medial side was measured using protractor. Angles of 90 ± 3° were accepted as the normal boundaries; less than 87° was considered varus, and more than 93° was considered valgus.

The mechanical axis of the lower extremity is defined as the hip‐knee‐ankle angle (HKA), which is, the medial angle formed by the mechanical axis of the femur and the mechanical axis of the tibia. The mechanical axis of the femur was defined as the connection between the center of the femoral head and the midpoint of the femoral component. The mechanical axis of the tibia are defined as connection between the center of the tibia prosthesis and the center of the ankle joint.

In a study by Bilgen *et al*., the mean value of the medial angle of the tibial prosthesis was 89.4% (89.17° when the EHLT was used as the distal marker and 88.57° when the second metatarsal was used as the distal marker), there was no significant difference in mean CTA between groups (*P* = 0.124); however, the authors reported that 69.8% of the cases in the two groups had <2° external and internal valgus, respectively, The number of tibial components with alpha angles in the normal range was significantly higher in the ERT group (*P* = 0.017)^7^ (Table 1).

**TABLE 1:** Coronal alignment of the tibial prostheses

Reference points		Mean age (years)	Follow‐up Time	Total number of patients	Evaluation of alignment		
Proximal	Distal				CTA^a^, mean ± standard deviation (range)	87°<CTA<93°	
Nishikawa *et al*.^30^							
Surgeon's subjective judgment using bony and soft tissue landmarks		74.4	Jan 2007 to Mar 2010	114	89.6° ± 1.8° (85.7° to 93.8°)	*n* = 102 (87.2%)	*P* < 0.05^b^
Proximal one‐third of the anterior border of tibia	Distal one‐third of the anterior border of tibia	76.8	Oct 2010 to Sep 2013	74	89.3° ± 1.6° (84.5°–92.7°)	*n* = 71 (95.9%)	
Sasanuma *et al*.^13^							
Center of the proximal tibia	Center of the ankle	75	Dec 2009 to Oct 2010	97	90.1° ± 2.2°	*n* = 85 (88%)	*P* = 0.97
Proximal one‐third of the anterior border	Distal one‐third of the anterior border of tibia	72	Oct 2010 to May 2012.	76	90.1° ± 2.5°	*n* = 64 (84%)	
Bilgen *et al*.^7^						88°<CTA<92°	
Not mentioned	Extensor hallucis longus tendon	68.3	2004 to 2006	47	89.17 ± 1.74° (84°–93°)	*n* = 42 (89.4%)	*P* = 0.017
	Second metatarsal	70.2	2006 to 2008	53	88.57 ± 2.11° (84°–93°)	*n* = 37 (69.8%)	

^a^ The coronal tibial component angle (CTA) is the medial angle between the mechanical axis of the tibia and the horizontal axis of the tibial tray. CTA was measured from bipedal‐stance, weight‐bearing, full‐length standing radiographs of the operatively treated limb

^b^ *P* < 0.05: the proportion of cases achieving ideal alignment by the two methods was significantly different between the two groups.

A study reviewing the alignment of postoperative tibial prostheses in a total of 212 knee replacements in 188 patients with the TAT as the reference point, found that 191 (90.1%) tibial prostheses were well‐aligned. There were five postoperative valgus osteotomies and 16 postoperative internal valgus osteotomies. Of the 196 knees with internal rotation deformity preoperatively, 17 (8.7%) had poor force lines of the prosthesis postoperatively; the caliper test was not statistically correlated after correction (*P* = 0.096)^31^.

In contrast, the line connecting the medial third of the patellar tendon and the distal third of the tibia, and the line connecting the proximal third and distal third of the tibia, had the smallest interval of angular variation from the mechanical axis with a standard deviation of 0.78 and an angular range of −1.88° to 1.98°. All were within an acceptable range of 3°. when the latter method was used that showed a greater inter‐individual variation in the angular range of −3.5° to 3.1°. Three groups of 99% of the knee mechanical axes were within 3° of each other: the medial one‐third of the patellar tendon and the anterior one‐third of the distal tibial tuberosity, the tibial tuberosity apex and the distal one‐fourth of the tuberosity, and the proximal one‐third and distal one‐fourth of the anterior tibial tuberosity^25^.

In a clinical trial, Sasanuma *et al*. compared the accuracy of proximal tibial osteotomy using a line connecting the proximal and distal third of the anterior tibial margin, and the traditional method (a line connecting the proximal tibia to the center of the ankle). In terms of coronal alignment, the target angle (with ±3° deviation) was achieved in 84% and 88% of cases, respectively. The postoperative tibial prosthesis angle and the lower extremity force line angle were not significantly different in the two methods (*P* = 0.97)^13^ (Table 1).

In a similar clinical trial, Nishikawa *et al*. compared the line joining the proximal third and distal third of the anterior tibial margin (method A) as a reference line for the coronal plane mechanical axis with the traditional method (method B). The mean coronal tibial prosthesis angle (CTA) was 89.6° ± 1.8° (85.7°–93.8°) for method A *vs* 89.3° ± 1.6° (84.5°–92.7°) for method B. The mean HKA angle was 179.3° ± 3.6° (168.4°–187.6°) for method A *vs* 178.5° ± 3.1° (171.8°–185.5°) for method B. The mechanical axis angles did not differ between the two groups, but the proportion of cases achieving ideal alignment by the two methods was significantly different: 87.2% (102/117) with method B *vs* 95.9% (71/74) with method A; there was a significant difference in ideal CTA between the two groups^30^ (Table 1).

In a study by Tadashi *et al*., the author used 3D imaging software to analyze the angle of the tibial crest deviating from the mechanical axis on the coronal plane, and found that the average angle of the tibial crest‐tibial mechanical axis was 0.7° ± 0.9°(1.7° – 3.8° varus) on the coronal plane, and 98% (100% female) of the patients had the tibial crest 3° away from the mechanical axis^28^.

## Discussion

A study by Nishikawa *et al*. proved that the proximal one‐third and the distal one‐third of the anterior tibial border were reliable landmarks for the tibial extramedullary alignment guide in TKA^30^. In a clinical trial, Sasanuma *et al*. proved that the proximal and distal one‐third of the tibia provides good coronal alignment and better accuracy for the posterior tibial slope compared with the conventional method^13^. In a study by Bilgen *et al*., the EHL tendon is a reliable anatomical landmark to use with extramedullary guide systems and improves coronal alignment^7^. Hino *et al*. proved that using EHLT results in more accurate lower limb alignment than previously reported reference points^8^. The fibular shaft axis does not appear to be a reliable intra‐operative land mark among patients with osteoarthritis who underwent conventional TKA^32^.

During TKA, poor alignment leads to uneven load distribution on the tibial platform, increased prosthetic subsidence, and accelerated polyethylene wear^33^. Valgus alignment produces greater strain and bone fatigue^3^. Inversion of the tibial platform by >3.0° leads to overload on the cancellous bone of the medial tibia and is the most common cause of TKA failure^34^. Kinematic alignment is defined as the axis of knee flexion and extension not perpendicular to the mechanical axis after placement of the femoral and tibial components. The femoral and tibial components are repositioned according to this anatomical axis^35^. However, in studies examining motion alignment, the patellofemoral and tibiofemoral peak contact stresses were increased by 200% and 270%, respectively^36^.

Ritter *et al*. found that tibiofemoral prosthesis implantation with internal rotation and femoral prosthesis implantation with >8° external rotation were associated with increased rates of revision surgery. If the femoral implant is poorly aligned, the revision rate is high even when the tibial implant alignment is corrected to achieve neutral alignment of the entire limb^37^.

During placement of the prosthesis, the surgeon needs to consider alignment in the coronal, sagittal, and axial planes. Coronal alignment is mainly achieved by adjusting the ankle clamp and using the TAT or EHLT to represent the center of the ankle. The method relies on the surgeon's subjective judgment of whether or not the extramedullary positioning bar is aligned coronally with the mechanical axis. Coronal alignment requires the transverse axis of the prosthesis to be perpendicular to the mechanical axes of the femur and tibia. This places the prosthesis in the centre of the lower limb force line.

In the sagittal plane, the posterior tibial inclination angle is roughly determined by the surgeon manually adjusting the distance between the extramedullary positioning rod and tibial anterior margin. Reference to the long axis of the fibula and the anterior tibial cortex has also been proposed^38^. Deviations in the angle of osteotomy may occur throughout the osteotomy process and result in poor alignment of the prosthesis during placement. Sagittal alignment is usually necessary to maintain a reasonable posterior angle of inclination. Both the physiologic posterior angle of the tibial platform and the type of prosthesis must be taken into consideration. The significance of the posterior inclination is to maintain the tension of the cruciate ligament and to allow the femur to rotate and roll properly, relative to the tibial platform during lower extremity motion, to facilitate knee flexion. However, there is no standard posterior inclination angle for tibial osteotomies.

The inter‐epicondylar axis of the femur is the mechanical axis of knee flexion and extension. It can be used to determine the internal and external rotation of the femoral prosthesis, and with regard to axial rotational alignment, it is now generally accepted that the line connecting the medial edge of the tibial tuberosity to the midpoint of the posterior cruciate ligament and the curved bend of the anterior edge of the tibia are reliable reference lines for rotational alignment^39^.

Despite improvements in surgical techniques and surgical instruments, target alignment is achieved in only 70%–80% of patients with the use of extramedullary guides^40^. Inaccurate osteotomy can result from various factors, including individual variations in osseous structures and the surgeon's subjective judgment errors during positioning.

Computer navigation techniques are increasingly being used to improve alignment during TKA. Some reports comparing accuracy of postoperative prosthesis alignment with conventional TKA and computer‐assisted surgery have shown smaller deviations from coronal tibiofemoral alignment and fewer outliers (more than ±3° deviation) with the latter^41^. However, the long‐term efficacy of navigation TKA in improving alignment accuracy is has not yet been established.

Because the tibia exhibits physiologic torsion—with the degree of torsion varying from individual to individual—many patients with knee deformities also have foot deformities. In patients with ankle deformity, the mechanical axis may not be correctly identified when the second metatarsal or a point 3–5 mm medial to the center of the ankle are used as the reference. Precise positioning of points at the anterior edge of the tibia (one‐third and one‐quarter of the full length of the tibia) is also difficult during surgery.

In patients with end‐stage osteoarthritis in TKA, valgus knee deformity is related to hindfoot inversion position and valgus knee deformity to hindfoot valgus position. Most of the compensation for deformities from the hindfoot to the knee occur in the subtalar joint. Therefore, when TKA is considered in patients with knee arthritis, the surgeon should carefully look for the presence of any hindfoot deformity^17^. When this is present, tendons around the second metatarsal and ankle joint may be affected. Hino *et al*. found that tendon position is not related to bunion angle, forefoot deformity index, and knee valgus angle,^8^ but there are few other reports in the literature, and so further research is necessary.

Previous studies have tended to focus on the ankle joint and did not consider the knee and ankle joints as a whole. The actual position of the ankle joint during the TKA procedure was not simulated. The actual relationship between the center of the ankle and the tendons during surgery may be inaccurate. The use of the tendon as a distal reference point is also susceptible to differences in patient body mass index and ankle fat thickness.

Osteotomy deviations occur due to anatomical differences in the tibia or the soft tissues, or due to inconsistencies in the ankle position, making it impossible to have a standardized osteotomy reference point. Therefore, it is important to find a reliable reference axis for the coronal alignment of the tibial osteotomy applied during surgery. It is also advisable to use multiple anatomical landmarks as references to reduce errors in the alignment of components.

### Prospects

Osteoarthritis of the knee can cause changes in the alignment of the lower extremity in all planes. Severe knee deformity results in limited flexion and extension and may lead to other lower extremity deformities. Therefore, the key to successful knee replacement is to ensure proper osteotomy volume and soft tissue balance. The amount of osteotomy directly affects the position of the joint line and the balance of flexion and extension. Excessive osteotomy can result in unstable flexion, while too little osteotomy can result in a limitation of extension. Restoration of the anatomical alignment in various planes, accurate osteotomy volumes in the medial and lateral tibial plateau, and accurate reconstruction of the lower extremity force line are key to maintaining the longevity of the prosthesis. As surgical techniques improve, surgeons seek better post‐replacement knee function. However, studies published to date have not been able to conclusively prove the superiority of any specific alignment method; therefore, multiple alignment methods continue to be used in clinical practice. More clinical trials and imaging data are needed to identify the best alignment method for accurate coronal osteotomy. As the theories of force alignment continue to advance, different distal reference points can be selected based on preoperative measurements of the internal and external valgus angles, and can be verified intraoperatively by force line rods. In our opinion, use of combinations of different reference points on the lower extremity force line will help achieve the ideal lower extremity force line angle.

## References

[1] Gandhi R , Dhotar H , Razak F , Tso P , Davey JR , Mahomed NN . Predicting the longer term outcomes of total knee arthroplasty. Knee, 2010, 17: 15–18. 19589683 10.1016/j.knee.2009.06.003

[2] Fang DM , Ritter MA , Davis KE . Coronal alignment in total knee arthroplasty: just how important is it?. J Arthroplasty, 2009, 24: 39–43. 19553073 10.1016/j.arth.2009.04.034

[3] Berend ME , Ritter MA , Meding JB , *et al*. Tibial component failure mechanisms in total knee arthroplasty. Clin Orthop Relat Res, 2004, 428: 26–34. 10.1097/01.blo.0000148578.22729.0e 15534515

[4] Choong PF , Dowsey MM , Stoney JD . Does accurate anatomical alignment result in better function and quality of life? Comparing conventional and computer‐assisted total knee arthroplasty. J Arthroplasty, 2009, 24: 560–569. 18534397 10.1016/j.arth.2008.02.018

[5] Teter KE , Bregman D , Colwell CW Jr . Accuracy of intramedullary versus extramedullary tibial alignment cutting systems in total knee arthroplasty. Clin Orthop Relat Res, 1995, 321: 106–110. 7497654

[6] Tsukeoka T , Tsuneizumi Y , Lee TH . Accuracy of the second metatarsal as a landmark for the extramedullary tibial cutting guide in total knee arthroplasty. Knee Surg Sports Traumatol Arthrosc, 2014, 22: 2969–2974. 25160475 10.1007/s00167-014-3254-4

[7] Bilgen O , Bilgen S , Ermutlu C , Goksel F , Salar N . The extensor hallucis longus tendon as the distal reference point in total knee arthroplasty and tibial alignment. Acta Orthop Traumatol Turc, 2014, 48: 271–275. 24901916 10.3944/AOTT.2014.3155

[8] Hino M , Nakagawa S , Arai Y , *et al*. Extensor hallucis longus tendon is a new distal landmark for coronal tibial component alignment in total knee arthroplasty: a study of magnetic resonance imaging. J Orthop Surg (Hong Kong), 2020, 28: 2309499020912340. 32223512 10.1177/2309499020912340

[9] Schneider M , Heisel C , Aldinger PR , Breusch SJ . Use of palpable tendons for extramedullary tibial alignment in total knee arthroplasty. J Arthroplasty, 2007, 22: 219–226. 17275637 10.1016/j.arth.2006.04.023

[10] Rajadhyaksha AD , Mehta H , Zelicof SB . Use of tibialis anterior tendon as distal landmark for extramedullary tibial alignment in total knee arthroplasty: an anatomical study. Am J Orthop (Belle Mead NJ), 2009, 38: E68–E70. 19377655

[11] Tiftikci U , Serbest S , Burulday V . Can Achilles tendon be used as a new distal landmark for coronal tibial component alignment in total knee replacement surgery? An observational MRI study. Ther Clin Risk Manag, 2017, 13: 81–86. 28144149 10.2147/TCRM.S125551 PMC5248942

[12] Sugimura N , Ikeuchi M , Izumi M , Aso K , Ushida T , Tani T . The dorsal pedis artery as a new distal landmark for extramedullary tibial alignment in total knee arthroplasty. Knee Surg Sports Traumatol Arthrosc, 2014, 22: 2618–2622. 23455389 10.1007/s00167-013-2461-8

[13] Sasanuma H , Sekiya H , Takatoku K , Ajiki T , Hagiwara H . Accuracy of a proximal tibial cutting method using the anterior tibial border in TKA. Eur J Orthop Surg Traumatol, 2014, 24: 1525–1530. 24449002 10.1007/s00590-014-1415-2

[14] Akagi M , Asada S , Mori S , Matsushita T , Hashimoto K , Hamanishi C . Estimation of frontal alignment error of the extramedullary tibial guide on the bi‐malleolar technique: a simulation study with magnetic resonance imaging. Knee, 2012, 19: 836–842. 22507073 10.1016/j.knee.2012.03.006

[15] Todesca A , Garro L , Penna M , Bejui‐Hugues J . Conventional versus computer‐navigated TKA: a prospective randomized study. Knee Surg Sports Traumatol Arthrosc, 2017, 25: 1778–1783. 27306985 10.1007/s00167-016-4196-9

[16] Mizu‐uchi H , Matsuda S , Miura H , Higaki H , Okazaki K , Iwamoto Y . The effect of ankle rotation on cutting of the tibia in total knee arthroplasty. J Bone Joint Surg Am, 2006, 88: 2632–2636. 17142413 10.2106/JBJS.E.01288

[17] Norton AA , Callaghan JJ , Amendola A , *et al*. Correlation of knee and hindfoot deformities in advanced knee OA: compensatory hindfoot alignment and where it occurs. Clin Orthop Relat Res, 2015, 473: 166–174. 25024033 10.1007/s11999-014-3801-9 PMC4390938

[18] Golightly YM , Hannan MT , Dufour AB , Renner JB , Jordan JM . Factors associated with hallux valgus in a community‐based cross‐sectional study of adults with and without osteoarthritis. Arthritis Care Res (Hoboken), 2015, 67: 791–798. 25418024 10.1002/acr.22517 PMC4440851

[19] Tsukeoka T , Tsuneizumi Y , Lee TH . The effect of rotational fixation error of the tibial cutting guide and the distance between the guide and the bone on the tibial osteotomy in total knee arthroplasty. J Arthroplasty, 2013, 28: 1094–1098. 23528550 10.1016/j.arth.2012.12.008

[20] Hobson J , Bicknell C , Cheshire N . Dorsalis pedis arterial pulse: palpation using a bony landmark. Postgrad Med J, 2003, 79: 363. 10.1136/pmj.79.932.363-a PMC1742720 12840142

[21] Meade TW , Gardner MJ , Cannon P , Richardson PC . Observer variability in recording the peripheral pulses. Br Heart J, 1968, 30: 661–665. 5676935 10.1136/hrt.30.5.661 PMC487695

[22] Siston RA , Daub AC , Giori NJ , Goodman SB , Delp SL . Evaluation of methods that locate the center of the ankle for computer‐assisted total knee arthroplasty. Clin Orthop Relat Res, 2005, 439: 129–135. 16205151 10.1097/01.blo.0000170873.88306.56

[23] Asada S , Mori S , Inoue S , Tsukamoto I , Akagi M . Location of the ankle center for total knee arthroplasty. Knee, 2017, 24: 121–127. 27825939 10.1016/j.knee.2016.09.019

[24] He P , Zhu Q , Zhang Z , Zou X , Xu D . Relationship between the tibial mechanical axis and bony anatomical landmarks of the calf and foot as measured on radiographs obtained with a new laser‐calibrated position. J Xray Sci Technol, 2013, 21: 497–506. 24191987 10.3233/XST-130400

[25] Fukagawa S , Matsuda S , Mitsuyasu H , *et al*. Anterior border of the tibia as a landmark for extramedullary alignment guide in total knee arthroplasty for varus knees. J Orthop Res, 2011, 29: 919–924. 21259337 10.1002/jor.21335

[26] Cinotti G , Caruso E , Orsina L , La Torre G , Ripani FR . Mapping of the anterior tibial profile to identify accurate reference points for sagittal alignment of tibial component in total knee arthroplasty. Orthop Traumatol Surg Res, 2017, 103: 959–963. 28645703 10.1016/j.otsr.2017.05.018

[27] Cinotti G , Ripani FR , Sinno E , Sarti S , LaTorre G , Giannicola G . Revisiting the tibial crest as reference for the mechanical alignment of the tibial component in total knee arthroplasty: a cadaveric study on Caucasian tibiae. Musculoskelet Surg, 2020. 10.1007/s12306-020-00639-x 32002790

[28] Tsukeoka T , Lee TH , Tsuneizumi Y , Suzuki M . The tibial crest as a practical useful landmark in total knee arthroplasty. Knee, 2014, 21: 283–289. 23154034 10.1016/j.knee.2012.09.002

[29] Cinotti G , Sessa P , Rocca AD , Ripani FR , Giannicola G . Effects of tibial torsion on distal alignment of extramedullary instrumentation in total knee arthroplasty. Acta Orthop, 2013, 84: 275–279. 23594222 10.3109/17453674.2013.792032 PMC3715826

[30] Nishikawa K , Mizu‐uchi H , Okazaki K , Matsuda S , Tashiro Y , Iwamoto Y . Accuracy of proximal tibial bone cut using anterior border of tibia as bony landmark in total knee arthroplasty. J Arthroplasty, 2015, 30: 2121–2124. 26233701 10.1016/j.arth.2015.06.055

[31] Zhao MW , Tian H , Zeng L , Li BG , Zhang FL , Li LY . Evaluation and analysis of the tibial coronal alignment after total knee replacement with the extramedullary tibial cutting guided by the tibial tubercle and anterior tibial tendon in Chinese patients. Beijing Da Xue Xue Bao Yi Xue Ban, 2016, 48: 351–355. 27080295

[32] Kuroda Y , Ishida K , Matsumoto T , *et al*. Fibular axes are not a reliable landmark for tibial mechanical axes of osteoarthritic knees that underwent total knee arthroplasty. Knee Surg Sports Traumatol Arthrosc, 2015, 23: 3362–3367. 25079132 10.1007/s00167-014-3170-7

[33] Hollister AM , Jatana S , Singh AK , Sullivan WW , Lupichuk AG . The axes of rotation of the knee. Clin Orthop Relat Res, 1993, 290: 259–268. 8472457

[34] Wong J , Steklov N , Patil S , *et al*. Predicting the effect of tray malalignment on risk for bone damage and implant subsidence after total knee arthroplasty. J Orthop Res, 2011, 29: 347–353. 20882595 10.1002/jor.21221

[35] Burnett RS , Barrack RL . Computer‐assisted total knee arthroplasty is currently of no proven clinical benefit: a systematic review. Clin Orthop Relat Res, 2013, 471: 264–276. 22948522 10.1007/s11999-012-2528-8 PMC3528921

[36] Ishikawa M , Kuriyama S , Ito H , Furu M , Nakamura S , Matsuda S . Kinematic alignment produces near‐normal knee motion but increases contact stress after total knee arthroplasty: a case study on a single implant design. Knee, 2015, 22: 206–212. 25813759 10.1016/j.knee.2015.02.019

[37] Jeffery RS , Morris RW , Denham RA . Coronal alignment after total knee replacement. J Bone Joint Surg Br, 1991, 73: 709–714. 1894655 10.1302/0301-620X.73B5.1894655

[38] Shao JJ , Parker Vail T , Wang QJ , *et al*. Anatomical references for tibial sagittal alignment in total knee arthroplasty: a comparison of three anatomical axes based on 3D reconstructed CT images. Chin Med J (Engl), 2013, 126: 3840–3844. 24157142

[39] Saffarini M , Nover L , Tandogan R , *et al*. The original Akagi line is the most reliable: a systematic review of landmarks for rotational alignment of the tibial component in TKA. Knee Surg Sports Traumatol Arthrosc, 2019, 27: 1018–1027. 30203197 10.1007/s00167-018-5131-z

[40] Mahaluxmivala J , Bankes MJ , Nicolai P , Aldam CH , Allen PW . The effect of surgeon experience on component positioning in 673 press fit condylar posterior cruciate‐sacrificing total knee arthroplasties. J Arthroplasty, 2001, 16: 635–640. 11503124 10.1054/arth.2001.23569

[41] Blakeney WG , Khan RJ , Wall SJ . Computer‐assisted techniques versus conventional guides for component alignment in total knee arthroplasty: a randomized controlled trial. J Bone Joint Surg Am, 2011, 93: 1377–1384. 21915542 10.2106/JBJS.I.01321

